# A Novel Approach to Identifying Hibernating Myocardium Using Radiomics-Based Machine Learning

**DOI:** 10.7759/cureus.69532

**Published:** 2024-09-16

**Authors:** Bangkim C Khangembam, Jasim Jaleel, Arup Roy, Priyanka Gupta, Chetan Patel

**Affiliations:** 1 Nuclear Medicine, All India Institute of Medical Sciences, New Delhi, IND; 2 Nuclear Medicine, Institute of Liver and Biliary Sciences, New Delhi, IND

**Keywords:** 18f-fdg cardiac pet, artificial intelligence, hibernating myocardium, machine learning, myocardial perfusion imaging, myocardial viability, radiomics, texture analysis

## Abstract

Background

To assess the feasibility of a machine learning (ML) approach using radiomics features of perfusion defects on rest myocardial perfusion imaging (MPI) to detect the presence of hibernating myocardium.

Methodology

Data of patients who underwent 99mTc-sestamibi MPI and 18F-FDG PET/CT for myocardial viability assessment were retrieved. Rest MPI data were processed on ECToolbox, and polar maps were saved using the NFile PMap tool. The reference standard for defining hibernating myocardium was the presence of mismatched perfusion-metabolism defect with impaired myocardial contractility at rest. Perfusion defects on the polar maps were delineated with regions of interest (ROIs) after spatial resampling and intensity discretization. Replicable random sampling allocated 80% (257) of the perfusion defects of the patients from January 2017 to September 2022 to the training set and the remaining 20% (64) to the validation set. An independent dataset of perfusion defects from 29 consecutive patients from October 2022 to January 2023 was used as the testing set for model evaluation. One hundred ten first and second-order texture features were extracted for each ROI. After feature normalization and imputation, 14 best-ranked features were selected using a multistep feature selection process including the Logistic Regression and Fast Correlation-Based Filter. Thirteen supervised ML algorithms were trained with stratified five-fold cross-validation on the training set and validated on the validation set. The ML algorithms with a Log Loss of <0.688 and <0.672 in the cross-validation and validation steps were evaluated on the testing set. Performance matrices of the algorithms assessed included area under the curve (AUC), classification accuracy (CA), F1 score, precision, recall, and specificity. To provide transparency and interpretability, SHapley Additive exPlanations (SHAP) values were assessed and depicted as beeswarm plots.

Results

Two hundred thirty-nine patients (214 males; mean age 56 ± 11 years) were enrolled in the study. There were 371 perfusion defects (321 in the training and validation sets; 50 in the testing set). Based on the reference standard, 168 perfusion defects had hibernating myocardium (139 in the training and validation sets; 29 in the testing set). On cross-validation, six ML algorithms with Log Loss <0.688 had AUC >0.800. On validation, 10 ML algorithms had a Log Loss value <0.672, among which six had AUC >0.800. On model evaluation of the selected models on the unseen testing set, nine ML models had AUC >0.800 with Gradient Boosting Random Forest (xgboost) [GB RF (xgboost)] achieving the highest AUC of 0.860 and could detect the presence of hibernating myocardium in 21/29 (72.4%) perfusion defects with a precision of 87.5% (21/24), specificity 85.7% (18/21), CA 78.0% (39/50) and F1 Score 0.792. Four models depicted a clear pattern of model interpretability based on the beeswarm SHAP plots. These were GB RF (xgboost), GB (scikit-learn), GB (xgboost), and Random Forest.

Conclusion

Our study demonstrates the potential of ML in detecting hibernating myocardium using radiomics features extracted from perfusion defects on rest MPI images. This proof-of-concept underscores the notion that radiomics features capture nuanced information beyond what is perceptible to the human eye, offering promising avenues for improved myocardial viability assessment.

## Introduction

Nearly four decades ago, Rahimtoola coined the term "hibernating myocardium", which refers to a state of persistently impaired myocardial and left ventricular (LV) function at rest due to reduced coronary blood flow that can be partially or completely restored to normal if the myocardial oxygen supply-demand relationship is favorably altered, either by improving blood flow and/or by reducing demand. It likely results from a relatively uncommon response to reduced myocardial blood flow at rest whereby the heart downgrades its myocardial function to the extent that blood flow and function are once again in equilibrium, and as a result, neither myocardial necrosis nor ischemic symptoms are present [1,2]. The improvements in myocardial contractility, LV function, and clinical outcome upon successful revascularization have been evident in clinical trials [3-6]. The Surgical Treatment for Ischaemic Heart Failure (STICH) trial, however, has shown conflicting results. Although viability did predict outcomes, it was not independent of other parameters [7,8]. Critical caveats exist in the STICH trial concerning viability assessment and treatment randomization. Available data suggest that revascularization reduces mortality for patients with ischaemic LV dysfunction and a substantial volume of viable myocardium, and hence assessment of myocardial viability and hibernating myocardium could be incorporated into clinical decision-making for patients with coronary artery disease (CAD) and heart failure [9,10]. The importance of detecting hibernating myocardium lies in its potential to inform treatment decisions, provide prognostic value, optimize resource allocation, and ultimately improve patient outcomes in the context of ischemic heart disease and LV dysfunction. Current evidence and guidelines support the assessment of myocardial viability and hibernating myocardium to assist in clinical decision-making for patients with ischemic heart failure [11-15].

Imaging modalities for the assessment of myocardial viability in patients with ischemic LV dysfunction include dobutamine stress echocardiography, single photon emission computed tomography (SPECT), cardiac magnetic resonance imaging (CMR), and positron emission tomography (PET) [13,16]. Metabolic imaging with 2-[18F]-fluoro-2-deoxy-D-glucose (18F-FDG) PET is not limited by the presence of arrhythmias and can be used in critically ill patients, patients with advanced kidney disease, pacemakers, defibrillators, or metallic inserts. Among these imaging modalities, 18F-FDG PET has the highest sensitivity and is considered the gold standard for the assessment of myocardial viability and hibernating myocardium [14,17]. Typically, 18F-FDG PET imaging is performed in conjunction with a separate myocardial perfusion imaging (MPI) with either SPECT or PET perfusion traces. MPI images and metabolic 18F-FDG PET images are displayed side by side and qualitative or semiquantitative approaches are applied to the interpretation of perfusion-metabolism patterns [18,19].

With advances in computing technology of modern computers, there has been substantial growth in the utilization of machine learning (ML) in medical science, at least in the research setting, and nuclear cardiology is no exception. The current and emerging key implementations of ML in nuclear cardiology encompass the entire workflow of imaging procedures starting from image acquisition and dose reduction, image reconstruction, and attenuation correction to image segmentation, MPI diagnostic analysis, and outcome prediction such as MPI-derived risk stratification and prognostication. ML also has the potential to facilitate clinical and imaging big-data integration for personalized risk stratification by efficiently combining large numbers of clinical and quantitative imaging variables to optimize diagnostic or prognostic utility [20,21]. The majority of the clinical studies on ML in nuclear cardiology focused on MPI related to CAD diagnosis, risk assessment, prediction of revascularization and LV ejection fraction (EF), and prognostication [22-25]. The incorporation of radiomics into the ML approach is even more scarce with a handful of studies focusing on CAD diagnosis, risk stratification, and cardiac contractile pattern recognition [26-30]. To our knowledge, no studies utilize an ML approach with or without radiomics to detect hibernating myocardium. The current study aims to assess the feasibility of an ML approach based on rest MPI image radiomics to detect the presence of hibernating myocardium in a real-world setting.

## Materials and methods

Patient cohort

This is a single-center retrospective study with a non-interventional cross-sectional analytical study design. Data of patients who had a documented history of myocardial infarction (MI) and underwent 99mTc-sestamibi MPI and cardiac 18F-FDG PET for myocardial viability assessment were retrieved from January 2017 to January 2023. Studies that were considered suboptimal by a consensus of two experienced nuclear medicine physicians were excluded. The Institutional Ethics Committee approved the study (IEC-449/03.08.2023, RP-18/2023). The committee waived the need for written informed consent due to the retrospective nature of the study.

Image acquisition, processing, and display

All patients underwent rest-gated SPECT MPI in adherence with the American Society of Nuclear Cardiology (ASNC) guidelines [31]. Twenty-four to 30 mCi of 99mTc-sestamibi was injected intravenously at rest. Image acquisition was performed 45-60 min later. Patients were positioned supine with hands raised overhead and limb leads placed for ECG gating. Image acquisition was performed on a dual-head gamma camera (GE Infinia, Hawkeye 4, Waukesha, WI, USA). Images were acquired using a 15% window centered over the 140 keV photo peak of 99mTc with parallel hole, low energy, high-resolution collimator. ECG-gated SPECT imaging was performed with eight frames per cardiac cycle, using an 80% beat acceptance window. The acquisition was performed using step-and-shoot mode in body contoured orbit with a matrix size of 128x128 and the detector heads oriented at an angle of 90° to each other. Sixty projections (30 steps, 3° steps) of 20 s/projection were acquired over 180° from 45° right anterior oblique position to -135° left posterior oblique position. Image reconstruction was performed with Filtered Back Projection. The raw images were then postfiltered with a Butterworth filter (critical frequency 0.4, order 10). The SPECT projection images were reviewed in cine mode to assess patient movement, sources of potential attenuation artifacts, and gastric activity. The resulting transaxial image slices were reoriented to generate short-axis, vertical long-axis, and horizontal long-axis images, using vendor-provided software.

Cardiac 18F-FDG PET was performed in all the patients within one week of the SPECT MPI studies. All patients fasted for at least six hours and underwent rest non-gated cardiac 18F-FDG PET in adherence with the ASNC guidelines for myocardial viability study using an oral glucose load of 50 grams and insulin injection [18,19]. After intravenous injection of 10 mCi of 18F-FDG, patients were asked to rest in a quiet room. After an uptake period of 45-60 min, patients were kept in the supine position on the scanner table of a dedicated 128-slice time-of-flight (TOF) positron emission tomography-computed tomography (PET/CT) scanner with lutetium-based crystals (Discovery 710, GE Healthcare, Milwaukee, WI, USA). CT acquisition was performed using the following parameters: 120 kVp, auto mAs, 5 mm helical thickness, 0.6 s rotation time, 39.4 mm/rotation, 0.984 pitch, 2.5 mm slice thickness reconstruction, 15.7 cm field of view, and a matrix of 512×512. After the CT acquisition, emission PET data were acquired in 3D mode for 10 minutes in a one-bed position. PET data were acquired with a matrix size of 128×128 with a slice thickness of 3.3 mm. PET data were reconstructed with VUE Point FX (3D ordered subset expectation maximization with TOF and point spread function correction; two iterations and 24 subsets). CT data were used for attenuation correction. Reconstructed attenuation-corrected PET images, CT images, and fused PET and CT images were available for review in axial, coronal, and sagittal axes along with maximum intensity projection (MIP) and 3D cine mode functionality.

Image analysis and reference standard

The SPECT MPI data and cardiac 18F-FDG PET data were loaded and analyzed in ECToolbox (Emory Cardiac Toolbox, Emory University, Atlanta, GA, USA) on a Xeleris Workstation (GE Medical Systems; Waukesha, WI, USA). The perfusion-metabolism image sets were displayed side by side and visually assessed in the standard axes. Also, the gated myocardial function and contractility were assessed. Semiquantitative assessment for mismatch between the two image sets and viability assessment was performed using the "high-dose 99mTc-sestamibi/18F-FDG and dual tracer" toolbox databases of ECToolbox. Polar maps of the rest MPI data were saved using NFile PMap tool of ECToolbox for radiomics analysis. Two experienced nuclear medicine physicians analyzed and interpreted the images. The reference standard for defining hibernating myocardium was the presence of mismatched perfusion-metabolism defect with impaired myocardial contractility at rest.

Radiomics workflow

Data Collection, Curation, and Image Processing

SPECT MPI and cardiac 18F-FDG PET data of the eligible patients were retrieved from the Picture Archiving and Communication System (PACS) of the department into the local workstation. Only the studies of those patients with acceptable image quality upon consensus by two experienced nuclear medicine physicians were included and analyzed further.

For radiomics analysis, we used the Local Image Feature Extraction software package (LIFEx-7.4.0, LITO, Orsay, France) which is compliant with the Image Biomarker Standardization Initiative (IBSI) [32,33]. The polar maps were loaded on the LIFEx software. We used two-dimensional settings for the radiomics analysis. Image processing included 2D spatial resampling at 1 x 1 mm^2^ grid using Lagrangian polynomial interpolation (polynomial of degree 5) [34,35] and relative intensity discretization with number of grey levels 32.

Perfusion Defect Detection and Segmentation

Image segmentation was performed on LIFEx-7.4.0 wherein perfusion defects on the polar maps were delineated with 2D regions of interest (ROIs) using visual assessment. Delineation of the perfusion defects with ROIs was meticulously performed always in corroboration with the visual analysis of the MPI images on the ECToolbox on the Xeleris workstation which included analysis of the short-axis, vertical long-axis, horizontal long-axis cardiac slices, and polar map displays.

Radiomics Features Extraction and Pre-processing

**Figure 1:**
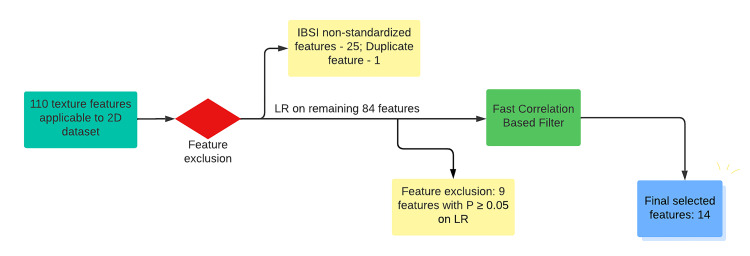
Steps of feature selection IBSI - Image Biomarker Standardization Initiative; LR - logistic regression

While extracting the radiomics features, we deliberately excluded, as a priori, those features which did not apply to the two-dimensional nature of the dataset used in the study. First and second-order radiomics features were extracted for each ROI using LIFEx-7.4.0. As a second step of feature selection, all those features that were not IBSI standardized were excluded. Univariate logistic regression analysis was then performed on the remaining features to select those with significant predictability for hibernating myocardium with a two-tailed p-value <0.05. Data pre-processing was performed on the radiomics features using Orange Data Mining Software 3.36.2, based on Python, in the form of feature normalization (standardization to μ=0, σ²=1), imputation, and a final feature selection step using the Fast Correlation-Based Filter (FCBF) to select the best ranked and stable features for training the ML algorithms [36, 37]. FCBF is an entropy-driven measure, which also detects data redundancy due to pairwise correlations between the features [38]. The steps of feature selection are depicted in Figure 1.

Training and Validation

Replicable random sampling was employed to allocate 80% of the perfusion defects (delineated by the ROIs) of the patients from January 2017 to September 2022 to the training set and the remaining 20% to the validation set. Thirteen supervised ML algorithms were trained with stratified five-fold cross-validation on the training set and validated on the validation set with hyperparameter optimization using manual search to yield optimal performance. A random classifier algorithm called "constant" which always predicted the majority class was used to select the ML algorithms for testing based on the Log Loss (cross-entropy loss) value [39] of the random classifier. Various performance metrics of the algorithms were assessed including area under the curve (AUC), classification accuracy (CA), F1 score, precision, recall, and specificity. The characteristics of the ML algorithms are detailed in (Appendix 1).

Model Testing

**Figure 2:**
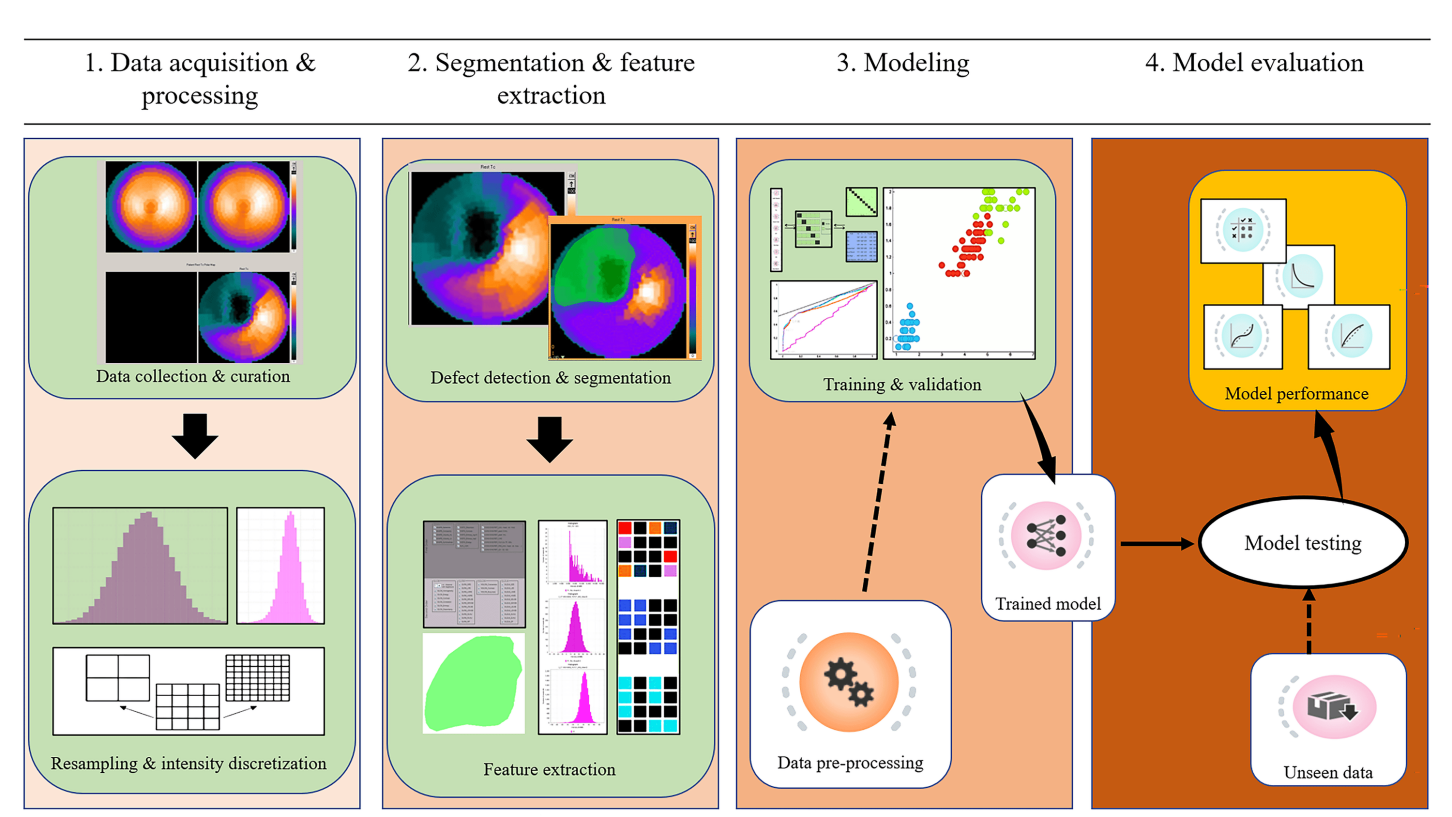
Radiomics and machine learning workflow

An independent dataset of perfusion defects (delineated by the ROIs) from 29 consecutive patients enrolled between October 2022 to January 2023 was used as the testing set for model evaluation. The ML algorithms with a Log Loss value less than that of the random classifier in the cross-validation and validation steps were selected for model testing on the testing set. The various performance metrics mentioned above were assessed and the classification performance of each selected model was also displayed in a confusion matrix which highlighted the actual vs predicted classifications in a cross-tabulation format with the correct predictions displayed on the diagonal. The radiomics and ML workflow are highlighted in Figure 2.

Statistical analysis

Descriptive statistics such as mean, standard deviation, frequencies, and percentages were used to describe the various patient characteristics and clinical profiles. Continuous variables were tested for normality with the Shapiro-Wilk test. Continuous variables between the groups (training and validation versus testing) were compared with the Student's t-test or Mann-Whitney U test as applicable. Categorical variables between the groups were compared with Pearson's Chi-squared test or Fischer's Exact test as appropriate. Univariate logistic regression was used as a step for feature selection to select the radiomics features that could significantly predict the presence of hibernating myocardium while the final feature selection step with FCBF was applied to the selected features by univariate logistic regression. Student's t-test with box plot representation was used to compare the final selected radiomics features stratified by the reference standard in the training set. To offer transparency and interpretability, the impact of the radiomics features on the model output for the prediction of hibernating myocardium was assessed with SHapley Additive exPlanations (SHAP) values [40,41]. A two-tailed p-value <0.05 was considered significant for the statistical analyses. All statistical analyses were performed with SPSS Statistics 26 (IBM Inc., Armonk, NY, USA) and MedCalc 19.6.4 (MedCalc Software, Ostend, Belgium). The development of ML models, training, validation, and testing was performed using Orange Data Mining Software 3.36.2, based on Python [36,37].

## Results

Patient characteristics

A total of 239 patients (214 males; mean age 56 ± 11 years) were enrolled in the study. Random sampling (80:20) of the perfusion defects (delineated by the ROIs) of the patients from January 2017 to September 2022 allocated 257 perfusion defects to the training set and the remaining 64 to the validation set. There was a total of 371 perfusion defects (321 in the training and validation sets and 50 in the testing set). Based on the reference standard, 168 perfusion defects had hibernating myocardium (139 in training and validation sets, 29 in testing set) while the remaining 203 did not contain hibernating myocardium (182 in the raining and validation sets, 21 in the testing set). The various patient characteristics and clinical profiles are detailed in Table 1.

**Table 1:** Patient characteristics and clinical profile SD - standard deviation; PCI - percutaneous coronary intervention; CABG - coronary artery bypass graft; SVD - single vessel disease; DVD - double vessel disease; TVD - triple vessel disease; CHB - complete heart block; CKD - chronic kidney disease; COPD - chronic obstructive pulmonary disease; CVA - cerebrovascular accident; LVEF - left ventricular ejection fraction

Characteristic	Training and Validation (N=210)	Testing (N=29)	p-value
Age in years (mean ± SD)	56 ± 11	53 ± 10	0.117
Sex			
Males	188 (89.5%)	26 (89.7%)	1.000
Females	22 (10.5%)	3 (10.3%)	
Perfusion defects			
Hibernating	139 (43.3%)	29 (48.7%)	0.052
Nonhibernating	182 (56.7%)	21 (51.3%)	
Diabetes mellitus			
Present	89 (42.4%)	11 (37.9%)	0.693
Absent	121 (57.6%)	18 (62.1%)	
Hypertension			
Present	110 (52.4%)	16 (55.2%)	0.844
Absent	100 (47.6%)	13 (44.8%)	
Family history			
Present	31 (14.8%)	6 (20.7%)	0.414
Absent	179 (85.2%)	23 (79.3%)	
Smoking			
Present	84 (40.0%)	8 (27.6%)	0.227
Absent	126 (60.0%)	21 (72.4%)	
Dyslipidemia			
Present	19 (9.0%)	2 (6.9%)	1.000
Absent	191 (91.0%)	27 (93.1%)	
Obesity			
Present	6 (2.9%)	0 (0.0%)	1.000
Absent	204 (97.1%)	29 (100.0%)	
PCI history			
Present	25 (11.9%)	5 (17.2%)	0.548
Absent	185 (88.1%)	24 (82.8%)	
CABG history			
Present	1 (0.5%)	0 (0.0%)	1.000
Absent	209 (99.5%)	29 (100.0%)	
Angiography			
SVD	13 (6.2%)	4 (13.8%)	0.250
DVD	50 (23.8%)	5 (17.2%)	
TVD	147 (70.0%)	20 (69.0%)	
Other comorbidities			
CHB	1 (0.5%)	0 (0.0%)	0.878
CKD	3 (1.4%)	0 (0.0%)	
COPD	2 (1.0%)	0 (0.0%)	
CVA	1 (0.5%)	0 (0.0%)	
Gout	1 (0.5%)	0 (0.0%)	
Hypothyroidism	8 (3.8%)	0 (0.0%)	
Silicosis	1 (0.5%)	0 (0.0%)	
None	193 (91.9%)	29 (100.0%)	
LVEF (%)	31 ± 8	28 ± 7	0.037

Feature selection

After excluding, as a priori, those features that did not apply to the two-dimensional nature of the dataset used in the study, a total of 110 first and second-order radiomics features were extracted for each ROI (Appendix 2). After excluding 25 IBSI non-standardized features and one duplicate feature (INTENSITY-BASED_50thIntensityPercentileIBSI:Y12H), univariate logistic regression was performed on the remaining 84 features to select the significant features for the prediction of hibernating myocardium. This resulted in the exclusion of nine radiomics features leaving 75 radiomics features. The final step of feature selection was executed with FCBF which resulted in the selection of 14 best-ranked and stable features, as detailed Appendix 3. Also, the box plot of one representative selected feature, and details of data preprocessing are highlighted in Appendix 2 and 4, respectively.

Training and validation

On stratified five-fold cross-validation on the training set, constant had a Log Loss value of 0.688. Out of the 13 ML algorithms trained with stratified five-fold cross-validation, four had a Log Loss value >0.688. These were k-Nearest Neighbours (kNN), Decision Tree, Naïve Bayes, and AdaBoost. Of the remaining nine ML algorithms, six had an AUC >0.800. These were Gradient Boosting (catboost (GB (catboost)), Random Forest, Support Vector Machine (SVM), Neural Network, Gradient Boosting Random Forest (xgboost (GB RF (xgboost)), and Gradient Boosting (scikit-learn (GB (scikit-learn)). The remaining three ML algorithms also had an AUC ≥0.790. These were Stochastic Gradient Descent (SGD), Logistic Regression, and Gradient Boosting (xgboost (GB (xgboost)). The various performance metrics of the ML algorithms on stratified five-fold cross-validation are detailed in Table 2.

**Table 2:** Training and validation ML - machine learning; AUC - area under the curve; CA - classification accuracy; GB - Gradient Boosting; kNN - k-Nearest Neighbours; SVM - Support Vector Machine; GB RF - Gradient Boosting Random Forest; SGD - Stochastic Gradient Descent; Constant - random classifier

Stratified 5-fold cross-validation							
ML algorithm	AUC	CA	F1 score	Precision	Recall	Specificity	Log Loss
GB (catboost)	0.817	0.755	0.732	0.717	0.748	0.761	0.535
Random Forest	0.815	0.763	0.740	0.725	0.757	0.768	0.531
kNN	0.815	0.743	0.708	0.721	0.696	0.782	0.781
SVM	0.810	0.735	0.712	0.694	0.730	0.739	0.543
Neural Network	0.810	0.732	0.701	0.698	0.704	0.754	0.550
GB RF ((xgboost)	0.804	0.732	0.714	0.683	0.748	0.718	0.666
GB (scikit-learn)	0.802	0.763	0.745	0.718	0.774	0.754	0.546
Decision Tree	0.796	0.767	0.746	0.727	0.765	0.768	1.691
SGD	0.796	0.724	0.687	0.696	0.678	0.761	0.591
Logistic Regression	0.792	0.724	0.695	0.686	0.704	0.739	0.615
GB (xgboost)	0.790	0.747	0.728	0.702	0.757	0.739	0.558
Naive Bayes	0.788	0.720	0.710	0.662	0.765	0.683	1.987
AdaBoost	0.630	0.634	0.591	0.591	0.591	0.669	12.633
Constant	0.500	0.553	0.000	0.000	0.000	1.000	0.688
Validation							
ML algorithm	AUC	CA	F1 score	Precision	Recall	Specificity	Log Loss
Random Forest	0.834	0.734	0.667	0.630	0.708	0.750	0.486
GB (xgboost)	0.830	0.750	0.680	0.654	0.708	0.775	0.511
kNN	0.816	0.781	0.720	0.692	0.750	0.800	0.503
GB (scikit-learn)	0.814	0.719	0.654	0.607	0.708	0.725	0.530
GB RF (xgboost)	0.810	0.750	0.680	0.654	0.708	0.775	0.667
Neural Network	0.807	0.734	0.653	0.640	0.667	0.775	0.512
GB (catboost)	0.798	0.734	0.667	0.630	0.708	0.750	0.534
Decision Tree	0.796	0.734	0.667	0.630	0.708	0.750	1.068
Logistic Regression	0.786	0.734	0.638	0.652	0.625	0.800	0.601
SVM	0.780	0.719	0.640	0.615	0.667	0.750	0.540
AdaBoost	0.779	0.766	0.727	0.645	0.833	0.725	8.095
SGD	0.779	0.703	0.596	0.609	0.583	0.775	0.549
Naive Bayes	0.757	0.703	0.642	0.586	0.708	0.700	2.327
Constant	0.500	0.625	0.000	0.000	0.000	1.000	0.672

On the validation step on the validation set, constant had a Log Loss value of 0.672. Decision Tree, AdaBoost, and Naïve Bayes had Log Loss values >0.672. Among the ten ML algorithms that had a Log Loss value <0.672 on the validation step, six had an AUC >0.800. These were kNN, Random Forest, GB (xgboost), GB (scikit-learn), GB RF (xgboost), and Neural Network. The remaining four ML algorithms had an AUC ≥0.779. The details of the performance metrics of the ML algorithms on the validation step are given in Table 2.

Feature importance

**Figure 3:**
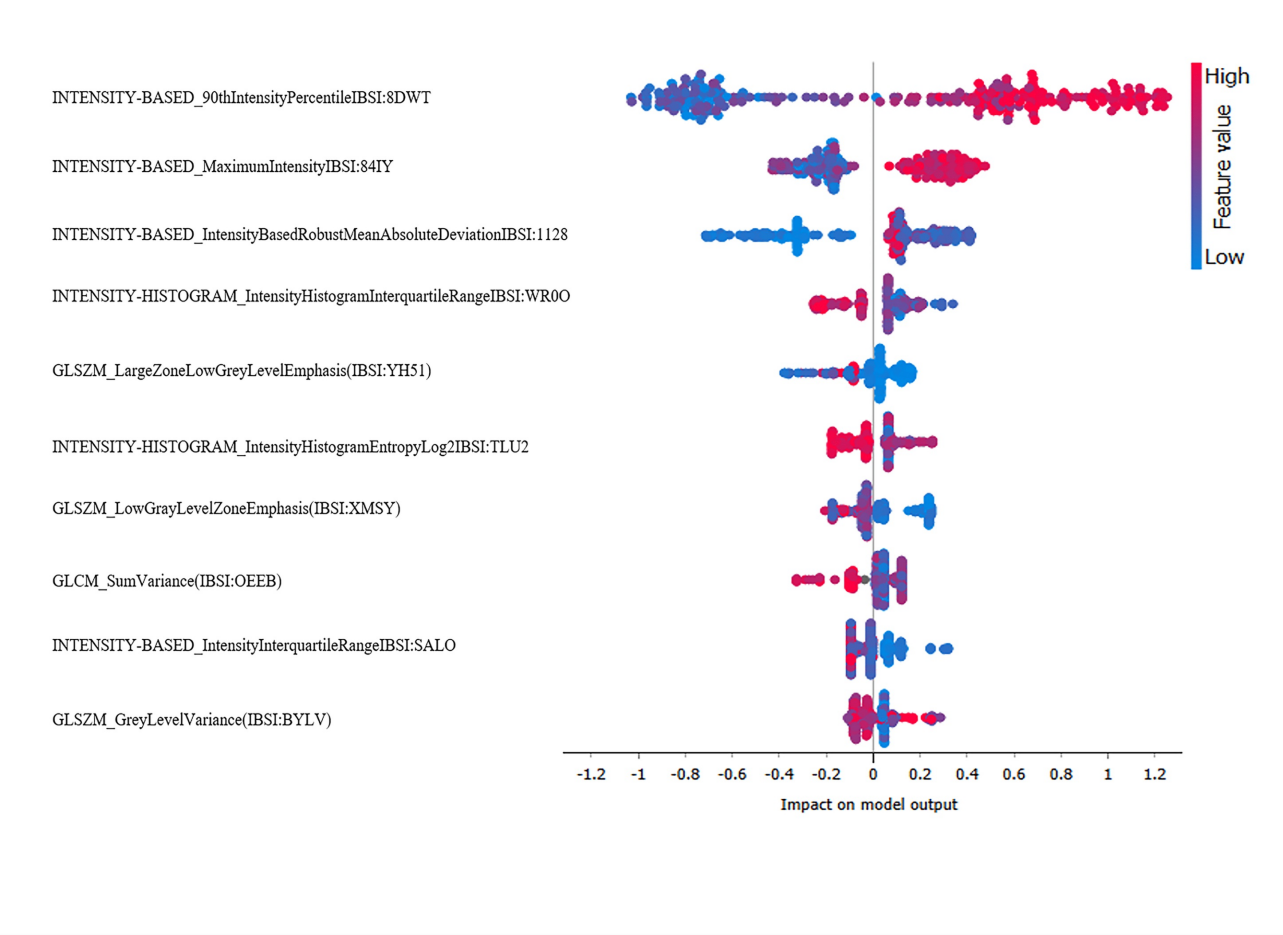
Beeswarm SHAP plot of GB (xgboost) model The plot illustrates the top texture features most significant to the model's predictions, with each feature represented by points indicating SHAP values along the horizontal axis for every region of interest. SHAP values quantify the influence of each feature on the model's prediction. SHAP values farther from the center signify a greater impact on the prediction for hibernating myocardium. SHAP values to the right from the center of the plot indicate that the feature value contributes positively to predicting hibernating myocardium, while values to the left indicate a negative impact. The colors of the points represent feature values, with red indicating higher values and blue indicating lower values, based on the full range of values for each feature in the dataset. SHAP - SHapley Additive exPlanations; GB - Gradient Boosting

To provide interpretability of the models' predictions, we employed beeswarm SHAP plots. The beeswarm SHAP plots were employed for the trained and validated nine ML algorithms (models) with a Log Loss <0.688 and 0.672 in the training and validation steps respectively (representative images in Figure 3, Appendix 5). Four models depicted a clear pattern of model interpretability based on the beeswarm SHAP plots. These were GB RF (xgboost), GB (scikit-learn), GB (xgboost), and Random Forest. The beeswarm plots of the remaining five models revealed a lack of interpretability as evidenced by the congregation of the SHAP values on or near the 'zero' line.

Model performance and generalization

**Figure 4:**
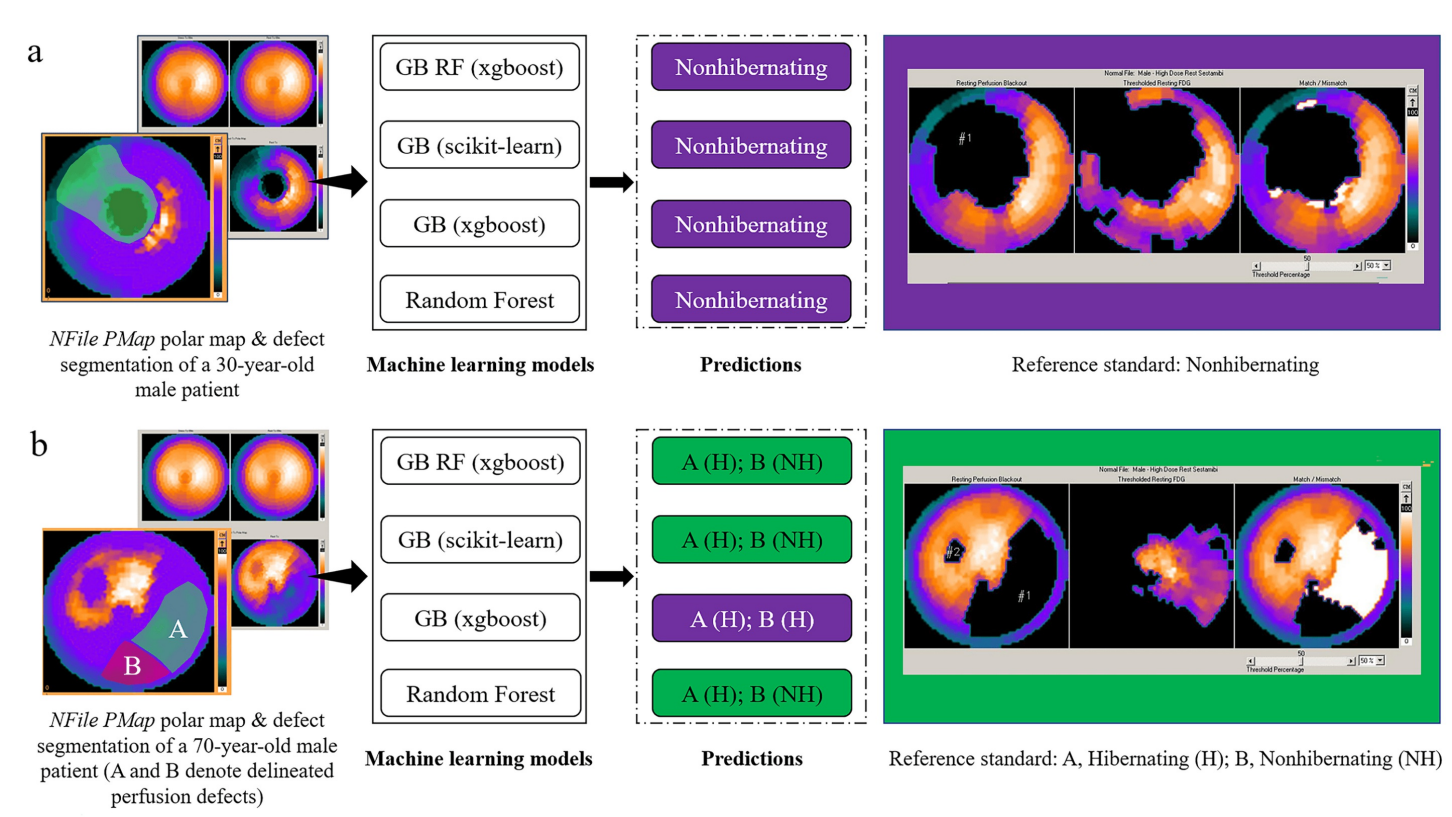
Classification performance of four machine learning models on the perfusion defects of two patients from the testing set (a) All four models correctly predicted the absence of hibernating myocardium in the perfusion defect of a 30-year-old male patient. (b) In a 70-year-old male patient with two perfusion defects, all four models correctly predicted the presence of hibernating myocardium in the perfusion defect 'A'; GB (xgboost) misclassified perfusion defect 'B' as harboring hibernating myocardium while the rest three models correctly classified it as having no hibernating myocardium. GB - Gradient Boosting; GB RF - Gradient Boosting Random Forest

**Figure 5:**
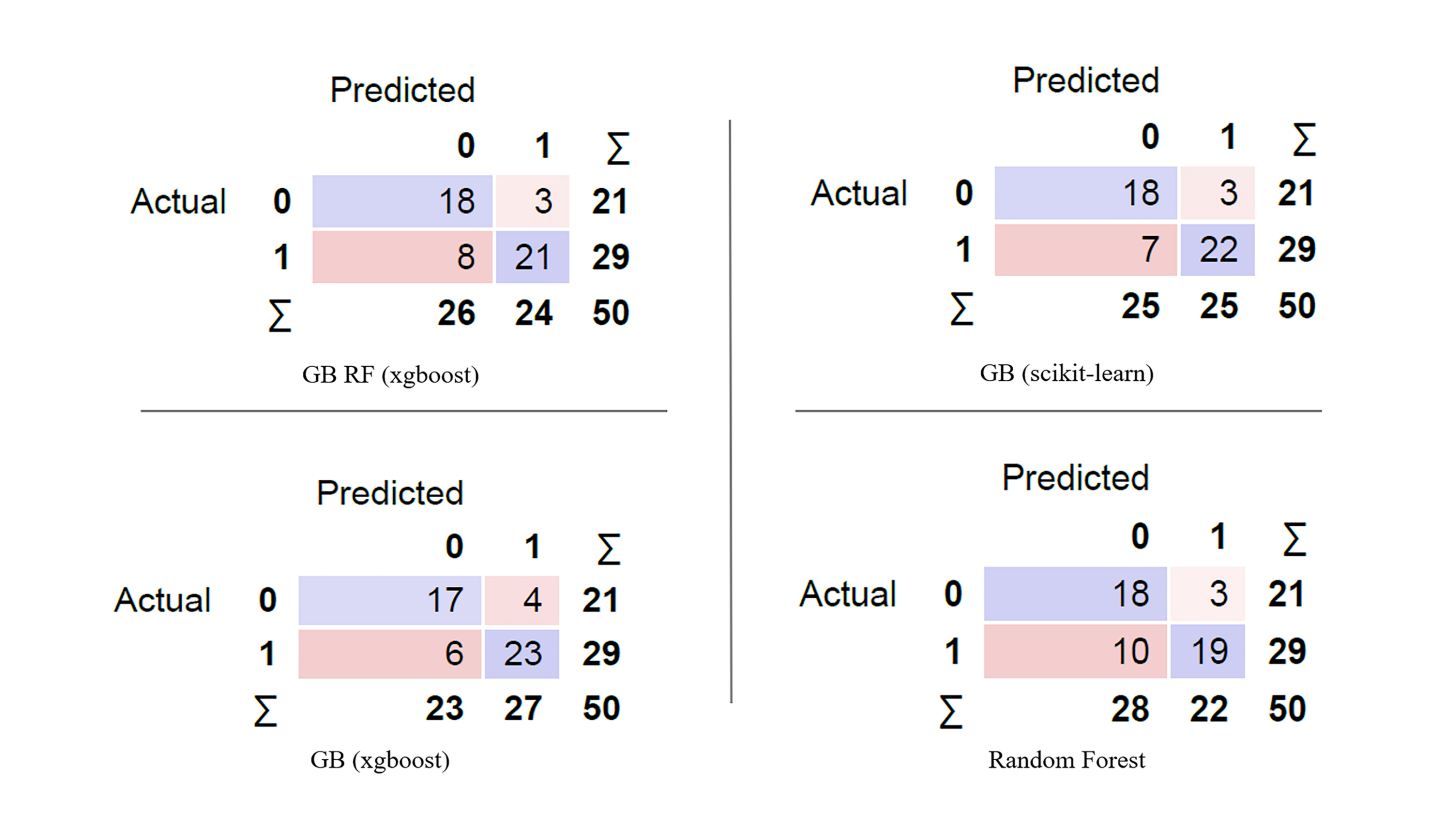
Confusion matrix of the predictions of four machine learning models on the testing set GB - Gradient Boosting; GB RF - Gradient Boosting Random Forest; 0 - Nonhibernating; 1 - Hibernating

The nine trained and validated ML models with a Log Loss value <0.688 and 0.672 in the training and validation steps respectively were selected for model evaluation and generalization. This was performed by testing the models' performance on the unseen testing set. All the nine models had AUC >0.800. The various performance metrics of these models are detailed in Table 3. The classification performance of the four ML models (with a clear pattern of model interpretability on the beeswarm SHAP plots) on the perfusion defects of two patients from the testing set is shown in Figure 4. Also, the predictions of these four models are displayed in the form of a confusion matrix in Figure 5.

**Table 3:** Model testing ML - machine learning; AUC - area under the curve; CA - classification accuracy; GB - Gradient Boosting; SVM - Support Vector Machine; GB RF - Gradient Boosting Random Forest; SGD - Stochastic Gradient Descent; Constant - random classifier

ML model	AUC	CA	F1 score	Precision	Recall	Specificity
GB RF (xgboost)	0.860	0.780	0.792	0.875	0.724	0.857
Logistic Regression	0.859	0.760	0.778	0.840	0.724	0.810
GB (scikit-learn)	0.848	0.800	0.815	0.880	0.759	0.857
GB (xgboost)	0.847	0.800	0.821	0.852	0.793	0.810
GB (catboost)	0.842	0.780	0.792	0.875	0.724	0.857
Random Forest	0.836	0.740	0.745	0.864	0.655	0.857
SGD	0.836	0.700	0.694	0.850	0.586	0.857
SVM	0.826	0.740	0.745	0.864	0.655	0.857
Neural Network	0.818	0.700	0.706	0.818	0.621	0.810
Constant	0.500	0.420	0.000	0.000	0.000	1.000

## Discussion

Radiomics is the extraction and analysis of quantitative features from medical images, transforming visual data into mineable high-dimensional information. This field aims to reveal nuanced relationships between image patterns and underlying pathophysiology, providing valuable insights for improved medical diagnosis and treatment planning [42]. ML is frequently employed in radiomics studies to handle the large and complex datasets generated from medical images. While its broad applicability extends to various medical conditions, ML and radiomics studies have seen the most significant development and extensive research in the field of oncology. In contrast, the domain of cardiac imaging in general and nuclear cardiology in particular has lagged in comparable developments and research endeavors. Indeed, a recent review of studies on cardiac imaging employing ML indicated that merely 15.9% involved the use of SPECT modality, while only 26.1% incorporated radiomics, predominantly relying on CT, MRI, or echocardiography [43]. This revelation highlights the importance of conducting more studies in the field. The current study aimed to assess the feasibility of an ML approach based on radiomics features of rest MPI images to predict the presence of hibernating myocardium, thereby offering a proof-of-concept demonstration in real-world conditions.

Wang et al. suggested that utilizing radiomics with LV tomograms from D-SPECT MPI holds the potential for aiding in the auxiliary diagnosis of myocardial ischemia in CAD patients [26]. Zhang et al. developed a radiomics ML model based on rest MPI PET images and could accurately predict coronary stenosis in patients with suspected CAD, thereby offering the feasibility of a non-invasive tool to improve risk stratification and clinical decision-making [27]. In another study, Sabouri et al. demonstrated the potential of ML models utilizing conventional quantitative and radiomic features from gated SPECT MPI to accurately predict LV contractile patterns, offering promising implications for improving patient selection for cardiac resynchronization therapy [28]. Degtiarova et al. demonstrated the feasibility of radiomics analysis based on visually normal rest MPI PET images in predicting reduced global myocardial flow reserve, thereby suggesting its potential application in improving the detection of diffuse myocardial perfusion abnormalities [29]. The study by Amini et al. revealed promising results for CAD risk classification using machine learning models built on MPI SPECT radiomics [30]. These studies underscore the promising intersection of ML and radiomics within the realm of nuclear cardiology. Notably, our study breaks new ground as the inaugural proof-of-concept endeavor utilizing ML and radiomics on rest MPI images for detecting hibernating myocardium. The compelling performances exhibited by our ML models in this preliminary investigation suggest exciting prospects for the future. Envisioning large-scale multicentric trials dedicated to this domain holds the potential to transform the paradigm of myocardial viability assessment. Incorporating rigorously validated radiomics ML models into standard imaging workflows holds great promise for optimizing the evaluation of myocardial viability, potentially streamlining and enhancing diagnostic accuracy in clinical practice. Automated radiomics analysis on rest MPI images could furnish probabilities and even quantifications of hibernating myocardium, presenting a monumental stride in workflow optimization.

The current study adopted a unique approach of selecting optimal and robust models for assessing model generalizability based on the Log Loss values of a random classifier. It considers the uncertainty of the prediction of each model based on how much it varies from the actual label [39]. Furthermore, our study delved into the crucial realm of transparency and interpretability of models, employing SHAP values, which are based on game theory. By assigning importance values to each feature in a model, SHAP values offer profound insights into model predictions [40,41]. Through the visualization tool of beeswarm SHAP plots, we not only elucidated the relative significance of various features but also unveiled the direction and magnitude of their influence on model predictions, fostering a deeper understanding of model dynamics. Our proof-of-concept study was conducted within real-world clinical settings. This approach mirrors real-world complexities, providing insights more representative than those obtained under controlled experimental conditions. This pragmatic approach underscores the relevance and applicability of our findings in clinical practice, paving the way for informed decision-making and enhanced patient care in real-world scenarios.

However, it is essential to acknowledge the study's limitations, with its retrospective design standing out as a primary constraint. Additionally, the relatively modest sample size, particularly for a study involving radiomics and ML, poses a limitation to the robustness of our findings. Moreover, the segmentation of perfusion defects for radiomics feature extraction relied on visual assessment, potentially introducing subjectivity. Despite these constraints, our proof-of-concept study offers valuable insights into the application of ML and radiomics for the evaluation of hibernating myocardium. To substantiate and validate our findings, large-scale multicentric prospective studies are essential. These studies will be crucial in fully elucidating the potential of these novel methodologies in clinical practice.

## Conclusions

Our study underscores the potential of using ML with radiomics features from rest MPI as a feasible and effective method for detecting hibernating myocardium. The results reveal that ML models can achieve commendable performance metrics, with an AUC exceeding 0.800 across various models, validating the robustness of this approach. This proof-of-concept highlights how radiomics features can capture subtle information beyond human perception, paving the way for enhanced myocardial viability assessments and opening new avenues for more accurate and personalized diagnostic approaches in clinical practice.

## Figures and Tables

**Figure 6:**
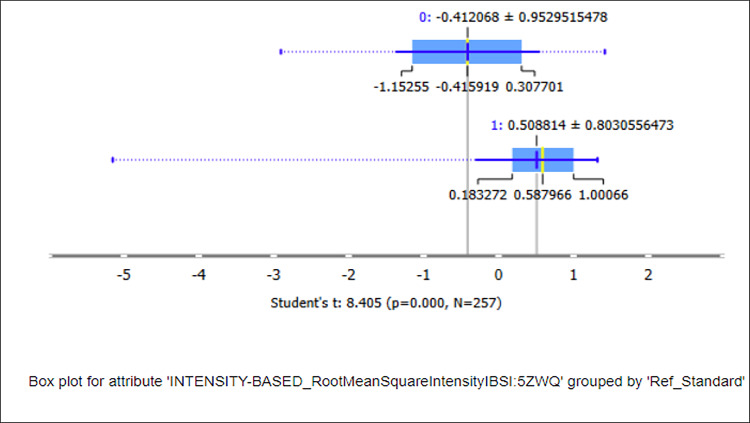
Appendix 4: Box plot of one representative selected radiomics feature stratified by reference standard Ref_Standard - reference standard

**Figure 7:**
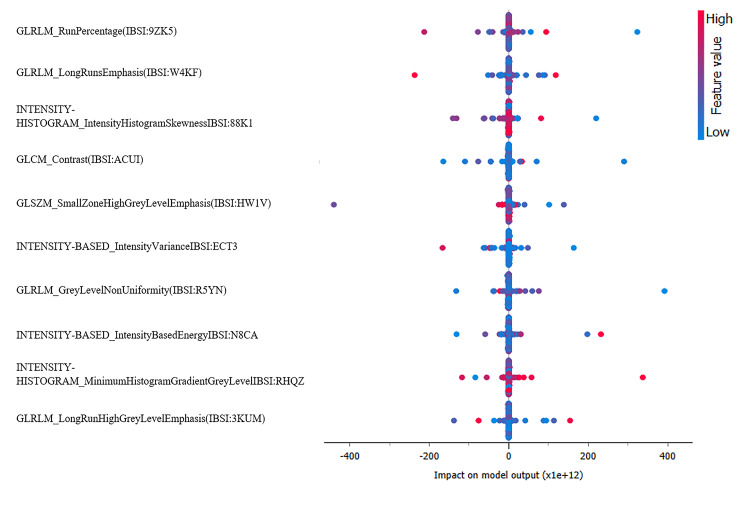
Appendix 5: Beeswarm SHAP plot of Neural Network model SHAP - SHapley Additive exPlanations

## Appendices

**Table 4:** Appendix 1: Details of hyperparameters and preprocessing of the machine learning algorithms [36] ML - machine learning; kNN - k-Nearest Neighbours; SVM - Support Vector Machine; RBF - Radial Basis Function; SGD - Stochastic Gradient Descent; RF - Random Forest Source: Demšar et al. [36]

ML algorithms	Hyperparameters	Preprocessing specific to each ML algorithm	Preprocessing for cross-validation
Constant (Random Classifier)			Normalize Features: Standardize to μ=0, σ²=1; Impute Missing Values: Average/Most frequent; Select Relevant Features: Fast Correlation Based Filter, Strategy (Fixed): 14
kNN	Number of neighbours: 21; Metric: Manhattan; Weight: Distance	Removes instances with unknown target values; Continuizes categorical variables (with one-hot-encoding); Removes empty columns; Imputes missing values with mean values; Normalizes the data by centering to mean and scaling to standard deviation of 1	Normalize Features: Standardize to μ=0, σ²=1; Impute Missing Values: Average/Most frequent; Select Relevant Features: Fast Correlation Based Filter, Strategy (Fixed): 14
Decision Tree	Pruning: at least 18 instances in leaves, at least 5 instances in internal nodes, maximum depth 100; Splitting: Stop splitting when majority reaches 95% (classification only); Binary trees: Yes		Normalize Features: Standardize to μ=0, σ²=1; Impute Missing Values: Average/Most frequent; Select Relevant Features: Fast Correlation Based Filter, Strategy (Fixed): 14
Random Forest	Number of trees: 64; Maximal number of considered features: unlimited; Replicable training: Yes; Maximal tree depth: 4 Stop splitting nodes with maximum instances: 7	Removes instances with unknown target values; Continuizes categorical variables (with one-hot-encoding); Removes empty columns; Imputes missing values with mean values	Normalize Features: Standardize to μ=0, σ²=1; Impute Missing Values: Average/Most frequent; Select Relevant Features: Fast Correlation Based Filter, Strategy (Fixed): 14
Logistic Regression	Regularization: Lasso (L1); C=6; class weights=No	Removes instances with unknown target values; Continuizes categorical variables (with one-hot-encoding); Removes empty columns; Imputes missing values with mean values	Normalize Features: Standardize to μ=0, σ²=1; Impute Missing Values: Average/Most frequent; Select Relevant Features: Fast Correlation Based Filter, Strategy (Fixed): 14
Naive Bayes		Removes empty columns; Discretizes numeric values to 4 bins with equal frequency	Normalize Features: Standardize to μ=0, σ²=1; Impute Missing Values: Average/Most frequent; Select Relevant Features: Fast Correlation Based Filter, Strategy (Fixed): 14
SVM	SVM type: SVM; C=1.0; ε=0.1; Kernel: RBF, exp(-0.01|x-y|²); Numerical tolerance: 0.001; Iteration limit: 100	Removes instances with unknown target values; Continuizes categorical variables (with one-hot-encoding); Removes empty columns; Imputes missing values with mean values; Normalizes dense and scales sparse data	Normalize Features: Standardize to μ=0, σ²=1; Impute Missing Values: Average/Most frequent; Select Relevant Features: Fast Correlation Based Filter, Strategy (Fixed): 14
Neural Network	Hidden layers: 7; Activation: tanh; Solver: Adam Alpha: 0.0001; Max iterations: 200; Replicable training: Yes	Removes instances with unknown target values; Continuizes categorical variables (with one-hot-encoding); Removes empty columns; Imputes missing values with mean values; Normalizes the data by centering to mean and scaling to standard deviation of 1	Normalize Features: Standardize to μ=0, σ²=1; Impute Missing Values: Average/Most frequent; Select Relevant Features: Fast Correlation Based Filter, Strategy (Fixed): 14
Gradient Boosting (scikit-learn)	Method: Gradient Boosting (scikit-learn); Number of trees: 20; Learning rate: 0.1; Replicable training: Yes; Maximum tree depth: 2; Fraction of training instances: 1.0; Stop splitting nodes with maximum instances: 2	Removes instances with unknown target values; Continuizes categorical variables (with one-hot-encoding); Removes empty columns; Imputes missing values with mean values	Normalize Features: Standardize to μ=0, σ²=1; Impute Missing Values: Average/Most frequent; Select Relevant Features: Fast Correlation Based Filter, Strategy (Fixed): 14
Extreme Gradient Boosting (xgboost)	Method: Extreme Gradient Boosting (xgboost); Number of trees: 30; Learning rate: 0.1; Replicable training: Yes; Maximum tree depth: 4; Regularization strength (Lambda): 8; Fraction of training instances: 1.0; Fraction of features for each tree: 1.0; Fraction of features for each level: 1.0; Fraction of features for each split: 1.0	Removes instances with unknown target values; Continuizes categorical variables (with one-hot-encoding); Removes empty columns; Imputes missing values with mean values	Normalize Features: Standardize to μ=0, σ²=1; Impute Missing Values: Average/Most frequent; Select Relevant Features: Fast Correlation Based Filter, Strategy (Fixed): 14
Gradient Boosting (catboost)	Method: Gradient Boosting (catboost); Number of trees: 26; Learning rate: 0.1; Replicable training: Yes; Maximum tree depth: 6; Regularization strength (Lambda): 3; Fraction of features for each tree: 1.0	Removes instances with unknown target values; Continuizes categorical variables (with one-hot-encoding); Removes empty columns; Imputes missing values with mean values	Normalize Features: Standardize to μ=0, σ²=1; Impute Missing Values: Average/Most frequent; Select Relevant Features: Fast Correlation Based Filter, Strategy (Fixed): 14
Extreme Gradient Boosting RF (xgboost)	Method: Extreme Gradient Boosting Random Forest (xgboost); Number of trees: 24; Learning rate: 0.1; Replicable training: Yes; Maximum tree depth: 6; Regularization strength (Lambda): 2; Fraction of training instances: 0.90; Fraction of features for each tree: 1.0; Fraction of features for each level: 1.0; Fraction of features for each split: 1.0	Removes instances with unknown target values; Continuizes categorical variables (with one-hot-encoding); Removes empty columns; Imputes missing values with mean values	Normalize Features: Standardize to μ=0, σ²=1; Impute Missing Values: Average/Most frequent; Select Relevant Features: Fast Correlation Based Filter, Strategy (Fixed): 14
SGD	Classification loss function: Logistic regression; Regression loss function: Squared Loss; Regularization: Lasso (L1); Regularization strength (α): 1e-05; Learning rate: Constant Initial learning rate (η_0_): 0.01; Shuffle data after each iteration: No	Removes instances with unknown target values; Continuizes categorical variables (with one-hot-encoding); Removes empty columns; Imputes missing values with mean values; Normalizes the data by centering to mean and scaling to standard deviation of 1	Normalize Features: Standardize to μ=0, σ²=1; Impute Missing Values: Average/Most frequent; Select Relevant Features: Fast Correlation Based Filter, Strategy (Fixed): 14
AdaBoost	Base estimator: tree; Number of estimators: 50; Learning rate: 1.0; Algorithm (classification): Samme.r; Loss (regression): Linear	Removes instances with unknown target values; Continuizes categorical variables (with one-hot-encoding); Removes empty columns; Imputes missing values with mean values	Normalize Features: Standardize to μ=0, σ²=1; Impute Missing Values: Average/Most frequent; Select Relevant Features: Fast Correlation Based Filter, Strategy (Fixed): 14

**Table 5:** Appendix 2: Radiomics features extracted using LIFEx-7.4.0 [32] GLCM - Gray-Level Cooccurrence Matrix; GLRLM - Gray-Level Run-length Matrix; NGTDM - Neighborhood Gray-Tone Difference Matrix; GLSZM - Gray-Level Size Zone Matrix Source: Nioche et al. [32]

First order features	Second order features
Morphological:	GLCM:
MORPHOLOGICAL_SurfaceArea(IBSI:C0JK)	GLCM_JointMaximum(IBSI:GYBY)
MORPHOLOGICAL_Compacity(IBSI:No)	GLCM_JointAverage(IBSI:60VM)
MORPHOLOGICAL_Compactness1(IBSI:SKGS)	GLCM_JointVariance(IBSI:UR99)
MORPHOLOGICAL_Compactness2(IBSI:BQWJ)	GLCM_JointEntropyLog2(IBSI:TU9B)
MORPHOLOGICAL_CentreOfMassShift(IBSI:KLMA)	GLCM_DifferenceAverage(IBSI:TF7R)
MORPHOLOGICAL_IntegratedIntensity(IBSI:99N0)	GLCM_DifferenceVariance(IBSI:D3YU)
Intensity-based:	GLCM_DifferenceEntropy(IBSI:NTRS)
INTENSITY-BASED_MeanIntensityIBSI:Q4LE	GLCM_SumAverage(IBSI:ZGXS)
INTENSITY-BASED_IntensityVarianceIBSI:ECT3	GLCM_SumVariance(IBSI:OEEB)
INTENSITY-BASED_IntensitySkewnessIBSI:KE2A	GLCM_SumEntropy(IBSI:P6QZ)
INTENSITY-BASED_IntensityKurtosisIBSI:IPH6	GLCM_AngularSecondMoment(IBSI:8ZQL)
INTENSITY-BASED_MedianIntensityIBSI:Y12H	GLCM_Contrast(IBSI:ACUI)
INTENSITY-BASED_MinimumIntensityIBSI:1GSF	GLCM_Dissimilarity(IBSI:8S9J)
INTENSITY-BASED_10thIntensityPercentileIBSI:QG58	GLCM_InverseDifference(IBSI:IB1Z)
INTENSITY-BASED_25thIntensityPercentileIBSI:No	GLCM_NormalisedInverseDifference(IBSI:NDRX)
INTENSITY-BASED_50thIntensityPercentileIBSI:Y12H	GLCM_InverseDifferenceMoment(IBSI:WF0Z)
INTENSITY-BASED_75thIntensityPercentileIBSI:No	GLCM_NormalisedInverseDifferenceMoment(IBSI:1QCO)
INTENSITY-BASED_90thIntensityPercentileIBSI:8DWT	GLCM_InverseVariance(IBSI:E8JP)
INTENSITY-BASED_StandardDeviationIBSI:No	GLCM_Correlation(IBSI:NI2N)
INTENSITY-BASED_MaximumIntensityIBSI:84IY	GLCM_Autocorrelation(IBSI:QWB0)
INTENSITY-BASED_IntensityInterquartileRangeIBSI:SALO	GLCM_ClusterTendency(IBSI:DG8W)
INTENSITY-BASED_IntensityRangeIBSI:2OJQ	GLCM_ClusterShade(IBSI:7NFM)
INTENSITY-BASED_IntensityBasedMeanAbsoluteDeviationIBSI:4FUA	GLCM_ClusterProminence(IBSI:AE86)
INTENSITY-BASED_IntensityBasedRobustMeanAbsoluteDeviationIBSI:1128	GLRLM:
INTENSITY-BASED_IntensityBasedMedianAbsoluteDeviationIBSI:N72L	GLRLM_ShortRunsEmphasis(IBSI:22OV)
INTENSITY-BASED_IntensityBasedCoefficientOfVariationIBSI:7TET	GLRLM_LongRunsEmphasis(IBSI:W4KF)
INTENSITY-BASED_IntensityBasedQuartileCoefficientOfDispersionIBSI:9S40	GLRLM_LowGreyLevelRunEmphasis(IBSI:V3SW)
INTENSITY-BASED_AreaUnderCurveCIVHIBSI:No	GLRLM_HighGreyLevelRunEmphasis(IBSI:G3QZ)
INTENSITY-BASED_IntensityBasedEnergyIBSI:N8CA	GLRLM_ShortRunLowGreyLevelEmphasis(IBSI:HTZT)
INTENSITY-BASED_RootMeanSquareIntensityIBSI:5ZWQ	GLRLM_ShortRunHighGreyLevelEmphasis(IBSI:GD3A)
Intensity-histogram:	GLRLM_LongRunLowGreyLevelEmphasis(IBSI:IVPO)
INTENSITY-HISTOGRAM_IntensityHistogramMeanIBSI:X6K6	GLRLM_LongRunHighGreyLevelEmphasis(IBSI:3KUM)
INTENSITY-HISTOGRAM_IntensityHistogramVarianceIBSI:CH89	GLRLM_GreyLevelNonUniformity(IBSI:R5YN)
INTENSITY-HISTOGRAM_IntensityHistogramSkewnessIBSI:88K1	GLRLM_RunLengthNonUniformity(IBSI:W92Y)
INTENSITY-HISTOGRAM_IntensityHistogramKurtosisIBSI:C3I7	GLRLM_RunPercentage(IBSI:9ZK5)
INTENSITY-HISTOGRAM_IntensityHistogramMedianIBSI:WIFQ	NGTDM:
INTENSITY-HISTOGRAM_IntensityHistogram10thPercentileIBSI:GPMT	NGTDM_Coarseness(IBSI:QCDE)
INTENSITY-HISTOGRAM_IntensityHistogram25thPercentileIBSI:No	NGTDM_Contrast(IBSI:65HE)
INTENSITY-HISTOGRAM_IntensityHistogram50thPercentileIBSI:No	NGTDM_Busyness(IBSI:NQ30)
INTENSITY-HISTOGRAM_IntensityHistogram75thPercentileIBSI:No	NGTDM_Complexity(IBSI:HDEZ)
INTENSITY-HISTOGRAM_IntensityHistogram90thPercentileIBSI:OZ0C	NGTDM_Strength(IBSI:1X9X)
INTENSITY-HISTOGRAM_IntensityHistogramStandardDeviationIBSI:No	GLSZM:
INTENSITY-HISTOGRAM_IntensityHistogramModeIBSI:AMMC	GLSZM_SmallZoneEmphasis(IBSI:5QRC)
INTENSITY-HISTOGRAM_IntensityHistogramInterquartileRangeIBSI:WR0O	GLSZM_LargeZoneEmphasis(IBSI:48P8)
INTENSITY-HISTOGRAM_IntensityHistogramMeanAbsoluteDeviationIBSI:D2ZX	GLSZM_LowGrayLevelZoneEmphasis(IBSI:XMSY)
INTENSITY-HISTOGRAM_IntensityHistogramRobustMeanAbsoluteDeviationIBSI:WRZB	GLSZM_HighGrayLevelZoneEmphasis(IBSI:5GN9)
INTENSITY-HISTOGRAM_IntensityHistogramMedianAbsoluteDeviationIBSI:4RNL	GLSZM_SmallZoneLowGreyLevelEmphasis(IBSI:5RAI)
INTENSITY-HISTOGRAM_IntensityHistogramCoefficientOfVariationIBSI:CWYJ	GLSZM_SmallZoneHighGreyLevelEmphasis(IBSI:HW1V)
INTENSITY-HISTOGRAM_IntensityHistogramQuartileCoefficientOfDispersionIBSI:SLWD	GLSZM_LargeZoneLowGreyLevelEmphasis(IBSI:YH51)
INTENSITY-HISTOGRAM_IntensityHistogramEntropyLog2IBSI:TLU2	GLSZM_LargeZoneHighGreyLevelEmphasis(IBSI:J17V)
INTENSITY-HISTOGRAM_AreaUnderCurveCshIBSI:No	GLSZM_GreyLevelNonUniformity(IBSI:JNSA)
INTENSITY-HISTOGRAM_UniformityIBSI:BJ5W	GLSZM_NormalisedGreyLevelNonUniformity(IBSI:Y1RO)
INTENSITY-HISTOGRAM_RootMeanSquareIBSI:No	GLSZM_ZoneSizeNonUniformity(IBSI:4JP3)
INTENSITY-HISTOGRAM_MaximumHistogramGradientIBSI:12CE	GLSZM_NormalisedZoneSizeNonUniformity(IBSI:VB3A)
INTENSITY-HISTOGRAM_MaximumHistogramGradientGreyLevelIBSI:8E6O	GLSZM_ZonePercentage(IBSI:P30P)
INTENSITY-HISTOGRAM_MinimumHistogramGradientIBSI:VQB3	GLSZM_GreyLevelVariance(IBSI:BYLV)
INTENSITY-HISTOGRAM_MinimumHistogramGradientGreyLevelIBSI:RHQZ	GLSZM_ZoneSizeVariance(IBSI:3NSA)
	GLSZM_ZoneSizeEntropy(IBSI:GU8N)

**Table 6:** Appendix 3: Final selected 14 radiomics features based on Fast Correlation Based Filter [36, 38] FCBF - Fast Correlation Based Filter; GLRLM - Gray-Level Run-length Matrix; NGTDM - Neighborhood Gray-Tone Difference Matrix; GLSZM - Gray-Level Size Zone Matrix Sources: Demšar et al. [36] and Yu et al. [38]

First order features	FCBF Score
INTENSITY-BASED_MeanIntensityIBSI:Q4LE	1.4422057587875264e-05
INTENSITY-BASED_MedianIntensityIBSI:Y12H	1.714793153928074e-05
INTENSITY-BASED_MinimumIntensityIBSI:1GSF	7.731745677206425e-06
INTENSITY-BASED_10thIntensityPercentileIBSI:QG58	1.0790955186758282e-05
INTENSITY-BASED_90thIntensityPercentileIBSI:8DWT	1.692119592116655e-05
INTENSITY-BASED_MaximumIntensityIBSI:84IY	0.17279198499084705
INTENSITY-BASED_IntensityBasedCoefficientOfVariationIBSI:7TET	7.310882371973579e-06
INTENSITY-BASED_IntensityBasedQuartileCoefficientOfDispersionIBSI:9S40	6.8195182783819925e-06
INTENSITY-BASED_RootMeanSquareIntensityIBSI:5ZWQ	1.4600385964342994e-05
Second order features	
GLRLM_LongRunLowGreyLevelEmphasis(IBSI:IVPO)	0.08579795507584674
NGTDM_Complexity(IBSI:HDEZ)	7.4461395127657634e-06
GLSZM_SmallZoneHighGreyLevelEmphasis(IBSI:HW1V)	7.791372294662323e-06
GLSZM_LargeZoneLowGreyLevelEmphasis(IBSI:YH51)	6.576407570273363e-06
GLSZM_NormalisedGreyLevelNonUniformity(IBSI:Y1RO)	7.6215738916300835e-06
